# What a city eats: Examining the dietary preferences of families living in communities at high risk for food insecurity

**DOI:** 10.1017/cts.2020.549

**Published:** 2020-10-19

**Authors:** Elaina Cummer, Claudia Loyola Amador, Kimberly Montez, Joseph A. Skelton, Brenda Ramirez, Scott Best, Rachel Zimmer, Deepak Palakshappa

**Affiliations:** 1 Wake Forest School of Medicine, Winston-Salem, NC, USA; 2 Department of Pediatrics, Wake Forest School of Medicine, Winston-Salem, NC, USA; 3 Help Our People Eat (HOPE) of Winston-Salem, Winston-Salem, NC, USA; 4 Department of Internal Medicine, Wake Forest School of Medicine, Winston-Salem, NC, USA

**Keywords:** Food insecurity, dietary preferences, meal preparation, mobile meal program, culturally relevant food

## Abstract

**Introduction:**

Food insecurity (FI) is the lack of consistent access to enough food for an active and healthy life. Community-based hunger relief programs often serve as emergency food sources for families with FI. However, these programs may not provide foods that diverse populations of people prefer. We sought to evaluate the dietary patterns and preferences of families living in food-insecure neighborhoods and utilizing a community-based hunger relief program, in order to improve the utilization of local nutritional programs.

**Methods:**

We examined the Help Our People Eat (HOPE) community-based mobile meal program. Free-listing interviews (*n* = 63) were conducted with English-(66%) and Spanish-speaking (34%) participants of the program. Participants were asked about FI risk, food preferences, and dietary behaviors at home.

**Results:**

The majority of participants (90%) had children in the household. About 60% reported not being able to afford the type of food they enjoyed. Most participants reported using stoves for cooking (80%). Participants overwhelmingly cooked with chicken, beef, and pork. The most common side dishes included potatoes, rice, and salad. Most participants reported no interest in cooking differently or learning new recipes.

**Conclusions:**

A common theme throughout interviews was that families prefer similar meals, but may prepare them differently based on the language spoken. Food preferences consisted of a high intake of carbohydrate-rich meals, perhaps because these foods may be cheaper and easier to access. Notably, new recipes and cooking methods were not a priority for these families, possibly due to the time and effort needed to learn them.

## Introduction

Food insecurity (FI) – the lack of consistent food for an active and healthy life – remains a major public health problem in the United States of America (USA) [1]. In 2018, 11.1% of US households, or over 35 million Americans were food insecure. Households with children are at higher risk of having FI: 13.9% of households with children were food insecure in 2018. Households with incomes near or below the federal poverty level and those headed by racial and ethnic minorities are also disproportionately vulnerable to FI [1–3]. While FI has decreased over the past decade, it remains problematic in North Carolina, which has the 10^th^ highest prevalence in the USA [1]. Winston-Salem, a medium-sized city located in Forsyth County in the Piedmont region of Northwest North Carolina, has an even higher FI rate: 16% for all households and 21% for households with children [4]. In both children and adults, FI negatively affects mental and physical health; however, children are particularly vulnerable due to its adverse effects on growth, behavior, and development [5–8].

FI is linked with poor dietary quality, including fewer fruits and vegetables, and lower nutrient intake [7]. Federal nutrition programs, such as the National School Meals Program (NSMP) and the Supplemental Nutrition Program (SNAP), provide more consistent access to nutritious food, improve dietary quality, and reduce FI for low-income households with children [9–15]. However, approximately 26% of food-insecure households in Winston-Salem do not qualify for these federal nutrition programs; furthermore, NSMP is unavailable when school is not in session [4]. Therefore, community-based hunger relief programs, such as mobile meal programs, have grown to further address local unmet needs. These organizations allow meals to be served or delivered in settings beyond schools, such as recreation centers, churches, and camps [16].

Although many studies have examined the nutrition-related behaviors and dietary preferences of participants who receive food from governmental nutrition programs, such as NSMP and SNAP, these studies have shown mixed results on the nutritional quality of food children receive [17]. There is sparse research available on mobile and community-based approaches in providing healthy and nutritious foods to diverse, low-income populations [18, 19]. To address the question of how community-based organizations can tailor the provision of healthy foods that participants living in diverse communities prefer, we undertook this exploratory study with the aim of understanding the dietary patterns, food preferences, and meal preparation methods of families at high risk for FI.

## Methods

### Study Setting and Population

We conducted a qualitative study of adults who utilize the Help Our People Eat (HOPE) (www.hopews.org) mobile meal program and assessed their dietary patterns, food preferences, and meal preparation methods. Adult participants were recruited from community locations that receive HOPE’s food resources, including weekend meals and produce delivery services.

Using the principles of community engagement, this study was designed, executed, and analyzed in close collaboration with our community partner, HOPE of Winston-Salem, which was founded in 2013 [20]. Every weekend, HOPE’s mobile meal program delivers free meals to children and fresh produce to families at risk for hunger, with the expressed goal of bridging children between school meals provided during the week and weekends when access to nutritious food may decline. Typical lunches provided for children include a sandwich, fruit/yogurt, milk/water, and a healthy treat. The produce offered to families varies every week based on supplier food donations, but occasionally includes non-produce items such as bread. This program partners with the local food bank, a variety of community gardens, and several food pantries in the area. HOPE delivers approximately 1100 healthy meals and 1500 lbs of fresh produce per week to 28 neighborhood sites consisting of apartment complexes, schools, community centers, and churches. These community sites are targeted by the poverty level based on census tract data and the number of children eligible for school breakfast/lunch at the neighborhood school (Table 1) [21]. All 28 sites are located in neighborhoods where at least 25% of the population lives below 200% of the federal poverty level, and 12 of those sites are in neighborhoods where 70% or more lives below 200% of the federal poverty level. Food deserts are defined by the USDA as census tract areas that qualify as low-income communities with low access to a supermarket, meaning they are more than one mile away in an urban area [22]. Twenty-three of the 28 sites are located in census tracts that qualify as food deserts [23]. All of the sites are within the Winston-Salem, North Carolina city limits. All adults who spoke either English or Spanish and presented to one of the HOPE sites were eligible to participate in this study.

**Table 1.** Population characteristics of the zip codes included in the study

	27103	27105	27107
Male	47%	47.9%	46.5%
Children (<18 years)	23.7%	25.5%	21.2%
Race Hispanic/Latino White Black/African American	14.6% 46.0% 34.8%	5.5% 72.4% 23.1%	12.3% 72.4% 13.3%
Percentage of adults (25 years of age and older) with a high school graduate/higher	87.5%	84%	90.6%
Percentage of adults (25 years of age or older) with a bachelor’s degree or higher	34.2%	19.9%	31.7%
Median household income	$44,311	$40,375	$49,407
Percent of individuals who live below 100% of the federal poverty level	21.7%	12.7%	14.2%

Data were obtained from the United States Census Bureau (2019): https://www.census.gov/quickfacts/fact/table/kernersvilletownnorthcarolina,walkertowntownnorthcarolina,winstonsalemcitynorthcarolina/PST04521920.

### Data Collection

**Fig. 1.**
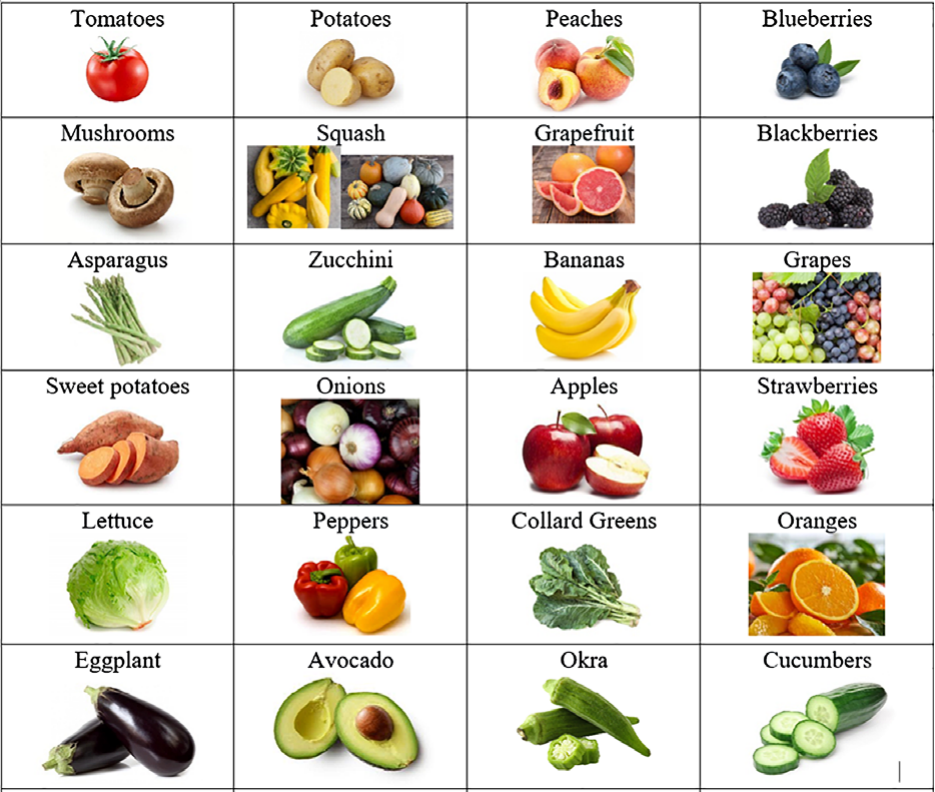
This graphic was shown when participants were asked about five favorite and least favorite produce items.

Through a detailed review of the literature and consultation with outside experts, we developed a free-listing interview guide consisting of 11 open-ended questions (Table 2) designed to elicit the food preferences and dietary behaviors of both adults and children in each household. These questions were pilot tested on five parents of children at risk for FI at the Downtown Health Plaza (DHP) Clinic of Wake Forest Baptist Health. DHP is a safety-net clinic that provides care to approximately 14,000 children from the same neighborhoods served by HOPE. The data from these five participants were not included in the final analysis. To evaluate fresh produce preferences, we showed participants a picture chart of various fruits and vegetables that have previously been offered by HOPE, and asked them to choose five items they used the most and five items they used the least at home (Fig. 1).

**Table 2.** Interview guide questions

• What is your favorite meal to cook/have cooked for you at home? o Tell me how you cook that meal. What foods or ingredients were used? o Do you use a stove, oven, or microwave?
• Are you able to afford the types of foods you like to eat?
• What are some other meals that you cook/are cooked regularly at home (≥once/month)?
• What are your child’s likes /dislikes about the prepared meals from HOPE that he/she receives? How about the fruits/vegetables? Does your family use the fruits/vegetables to cook?
• What are new meals or recipes that you want to learn how to cook at home? What are some ways you want to cook differently at home?
• What is your child’s favorite meal to eat that you cook at home?

Free-listing interviews were conducted between June and August 2019 by study team members (E.C., C.L.A., and K.M), who were all trained in qualitative interview techniques, using the guide. The technique of free listing involves asking open-ended questions to obtain a comprehensive and exhaustive list of items within a specific domain of interest, thus eliciting themes from all responses within a question. The purpose of free listing is to reduce the need for subsequent interviews [24, 25]. HOPE participants were approached at the time of meal and/or produce delivery along two different routes of the Sunday delivery program. Each route consisted of five–seven stops. Verbal consent was obtained prior to each interview in English or Spanish, dependent on the interviewee’s preferred language. Each interview was conducted in the participant’s preferred language (English or Spanish). Participants provided verbal responses while the interviewers recorded answers onto the interview guide, with each encounter lasting 5–10 min. Demographic information collected included primary language spoken at home, if there were children in the household, and FI risk (based on the response to the one question asking if participants were able to afford the types of food they enjoyed). To characterize the neighborhoods that participants were recruited from, we determined the percentage of males, households with children, race/ethnicity, and adults who graduated high school or had a bachelor’s degree, the median household income, and percent of individuals living below 100% of the federal poverty level in each zip code included based on data from the US Census Bureau (Table 1).

### Analysis

All interviews were transcribed and de-identified; data were analyzed using Microsoft Excel (Redmond, WA, USA) (2016). All responses were initially reviewed by a group of five study team members, which included the HOPE community partner. Qualitative data were analyzed within the cultural domains of interest: food preferences and behaviors. Responses were then listed by categories of main and side dishes. Using the constant-comparison method and group discussion, a common coding scheme was developed using the first five interviews, and codes were modified iteratively as needed [26]. Each transcript was then coded independently by two members of the team based on the coding scheme. Through an iterative process, the team regularly reviewed codes, identified emerging themes, and resolved discrepancies through consensus. Interviews continued until thematic saturation was reached. This study was deemed exempt from human subjects research by the Wake Forest University Health Sciences Institutional Review Board.

## Results

### Study Population Demographics

We conducted a total of 63 interviews at 12 HOPE sites within 3 different zip codes, predominately located in East and Southeast Winston-Salem. Of all participants, 68% (*n* = 43) were primarily English-speaking, and 32% (*n* = 20) were Spanish-speaking. Ninety percent (*n* = 57) of participants had children in the household. Fifty-seven percent (*n* = 36) of all respondents reported not being able to afford the type of food they enjoyed (Table 3).

**Table 3.** Study population characteristics

	*n* = 63	N (%)
Preferred language	English	43 (68%)
	Spanish	20 (32%)
Children in the household	Yes	57 (90%)
	No	6 (10%)
Able to afford the types of food they enjoyed	Yes	24 (38%)
	No	36 (57%)

### Families Preferred Chicken, Pasta, and Beef Dishes for Favorite Meals (Fig. 2)

When asked to share their household’s favorite meal to cook at home, participants responded with main dishes consisting of chicken (*n* = 34), most often fried or baked, followed by pasta (*n* = 18), beef (*n* = 15), and pork (*n* = 5). Overall, the most popular side dishes were potatoes (*n* = 10), rice (*n* = 7), rice and beans (*n* = 7), mixed vegetables (*n* = 7), and salad (*n* = 6). Spanish-speaking participants preferred main dishes containing chicken (*n* = 14) (notably chicken *mole*), beef (*n* = 3), and pasta (*n* = 3), with a side dish of rice and beans (*n* = 6). *Mole* is a traditional Mexican sauce that is used for a variety of meat dishes including chicken, turkey, and enchiladas [27]. Similarly, English-speaking participants reported primarily eating chicken (*n* = 23), pasta (*n* = 14), and beef (*n* = 13), although the chicken was most commonly fried (*n* = 10) in this group. When asked about children’s favorite meals, adults mentioned chicken (*n* = 24), pasta (*n* = 14), and beef (*n* = 6). A popular answer among participants was that the children eat “everything” or “whatever is on their plates.”

**Fig. 2.**
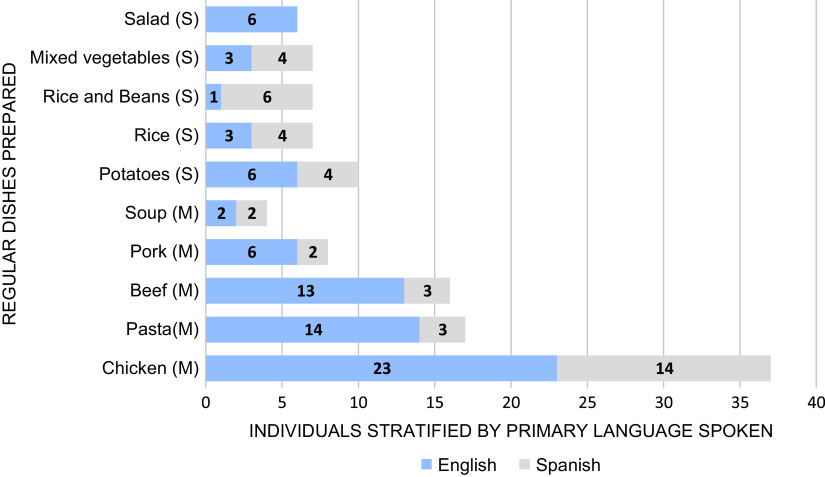
Top five meal preferences of Spanish- and English-speaking households. M, main dish; S, side dish.

### Adults Preferred Vegetables While Children Favored Fruit

**Fig. 3.**
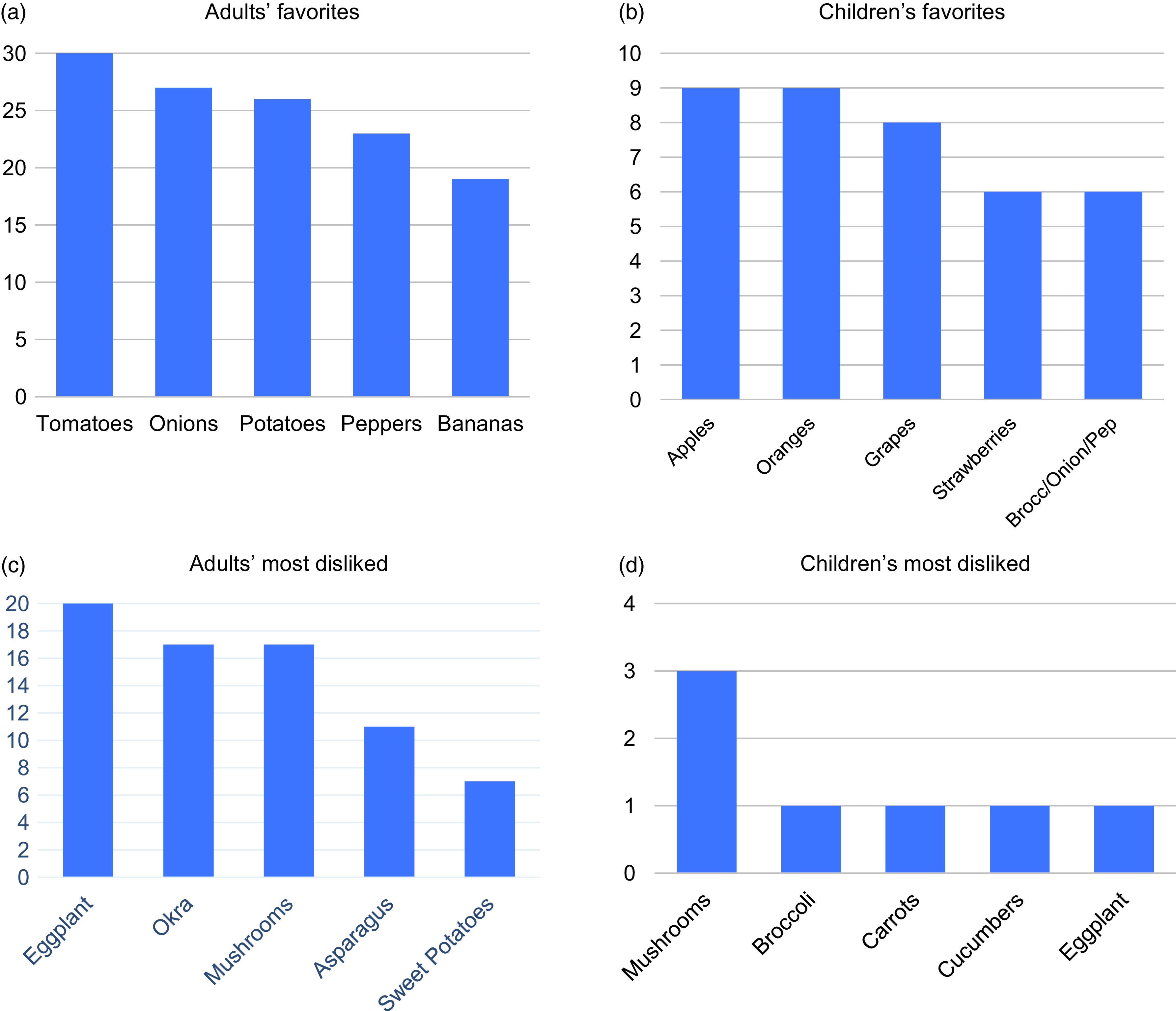
Most and least liked fresh produce preferences of participants interviewed. (N, number of respondents on y-axis, produce on x-axis). Answers stratified into adult and child preferences. Children’s preferences are primarily reported by adults. *Note:* Fig. 3(b) shows broccoli/onion/peppers as all preferred the same amount, consolidated in one bar.

We asked participants about their preferences for produce (Fig. 3a–d). Adults reported favoring tomatoes (*n* = 30), onions (*n* = 27), and potatoes (*n* = 26). The least favorite produce were eggplants (*n* = 20), mushrooms (*n* = 17), and okra (*n* = 17). English-speaking participants preferred onions (*n* = 20), peppers (*n* = 17), tomatoes (*n* = 15), and potatoes (*n* = 15), while Spanish-speaking participants liked tomatoes (*n* = 15), potatoes (*n* = 11), strawberries (*n* = 10), and mentioned more fruit in general. Okra, eggplant, and mushrooms were similarly disliked by all. Overall, children enjoyed more fruit than adults, especially apples (*n* = 9), oranges (*n* = 9), and grapes (*n* = 8).

### Adults Primarily Used Stoves at Home and Were Not Interested in New Ways of Cooking

When asked which appliances were most used to prepare meals, participants mentioned: stove (*n* = 60), oven (*n* = 10), and microwave (*n* = 5). There were no differences in the methods used to cook between English- and Spanish-speaking participants. A few families stated that they did not own a microwave. Participants were asked if they would like to learn any new meals or recipes and many responded with “none” (*n* = 36), or “did not answer” (*n* = 2). Some specified with: “cook with less salt/oil,” “cook with fresh produce,” and “how to eat as a diabetic.” When asked about specific ways that participants wanted to cook differently, 95% (*n* = 60) answered with “not sure” (*n* = 1), “none” (*n* = 30), or “did not answer” (*n* = 29).

## Discussion

In this study within a medium-sized southeastern city of 63 adults from diverse, low-income communities at high risk for FI, we found some dietary preferences in common: chicken, pasta, and beef were the most popular home-cooked dishes among adults and children alike. However, there were variations in preparation methods between English- and Spanish-speaking participants. Of all produce offered, adults enjoyed more vegetables, while reporting that children preferred more fruit. Lastly, families had little interest in learning new recipes or cooking methods at home and primarily used stoves.

Compared to individuals living in food-secure households, individuals living in food-insecure households have less healthy dietary patterns. Individuals in food-insecure households generally have a higher consumption of more palatable foods such as high-fat dairy and salty snacks, more red/processed meat, decreased frequency of fat-lowering behaviors, and less vegetable consumption [3, 7, 28, 29]. Our sample population had food preferences similar to what is described in the literature, with a high intake of calorie-dense foods and low consumption of vegetables. This could be because these foods, in comparison to fresh produce, may be cheaper, have longer shelf lives, provide more sustenance, and are easier to access within food deserts. Children have similar dietary patterns to adults in the same households, as seen in our study. Prior research has shown that children from food-insecure households have a higher likelihood of eating fast food, and can develop unhealthy eating patterns due to intermittent availability of food and increased stress levels [30–32]. While much of the food consumed was similar between English- and Spanish-speaking households, it was often prepared differently. Meat is typically held in high regard by many cultures, while other food items may have varying levels of significance [33]. It is important to understand the food preferences of particular groups of people, including culturally relevant foods (based on language, race/ethnicity, or religion, for example), so that community-based hunger relief programs, such as HOPE, can provide not only healthy, but culturally acceptable food.

Fresh produce preferences differed among adult and pediatric populations. We found that adults listed more vegetables, while children overwhelmingly liked fruit. This is consistent with literature showing that younger children rate fatty foods, sugary foods, and fruit the highest, and generally dislike vegetables [34, 35]. While the relationship of fruit and vegetable intake to weight has shown mixed results, various studies have shown an inverse association with intake and type II diabetes mellitus risk, visceral fat, liver fat, and insulin resistance in Hispanic/Latino children, thus making this a potentially important variable to evaluate in pediatric health [36, 37]. It has been shown that food preferences of parents, early introduction, and increased exposure of foods such as vegetables are important in increasing children’s preferences for such foods [38, 39]. Fruit and vegetable intake has also been directly correlated with its accessibility and availability at home, school, local markets, and the inclusion of children in cooking meals at home with their families [35, 40, 41]. In this manner, HOPE and other mobile meal programs may attempt to increase accessibility, availability, and visibility of fresh produce to encourage increased consumption by children and adults alike. Further research is needed to understand how community-based hunger relief programs can provide better access to fresh produce and encourage the intake of produce among food-insecure families.

One surprising finding in our study was that participants expressed little interest in learning new recipes or cooking methods. This may be due to financial constraints, time, and effort needed to learn to cook new recipes, and no guarantee that everyone in the household will eat a novel meal. In fact, one study showed that FI was associated with a lack of mealtime planning [42]. Other studies, such as Landers et al. [43], found that food-insecure families want to learn low-cost recipes such as soups based on canned stock or bouillon cubes, stir-fry, and one-dish skillet dinners that can be prepared in 30 min or less. Lack of supplies may also be a barrier to new methods of cooking, as literature has found that food-insecure households typically own fewer kitchen supplies than their food-secure counterparts [44]. The presence of food preparation supplies is associated with increased family meal frequency and child consumption of home-prepared dinners. Improving access to these supplies could be an important target in improving food-insecure families’ diets, as eating more family meals at home has been associated with the reduced caloric beverage and greater fruit and vegetable intake [45]. Literature shows that there may be areas of intervention such as teaching time-saving and cost-effective recipes, educating about meal planning, and providing more basic kitchen supplies to families. Further research is still needed to understand how families with limited budgets and time can eat healthier meals. Given the current literature and the results of our study, HOPE is encouraging local farmers to grow the produce that families reported they preferred and modifying the produce they provide to better address the needs of the families that utilize their services; furthermore, HOPE has made changes to the prepared meals in response to participant feedback such as ingredients in sandwiches and snacks. Additionally, a future development for HOPE is to begin offering cooking classes with recipes incorporating delivered produce that can generally be prepared within 30 min. Future studies and incentive programs could also consider distributing cooking utensils with related educational programming to encourage families to prepare more meals at home if they have successfully used the tools and successfully made a meal they enjoy.

There are several limitations to our study that should be acknowledged. First, although we tried to identify a diverse sample of adults from neighborhoods at high risk of FI, our small sample size may not be generalizable to other communities. Second, parents who agreed to participate in the study may not be representative of the populations in these neighborhoods due to selection bias. Third, because we were conducting interviews at the time participants were receiving services at HOPE, we limited the number of demographics collected for each participant to reduce the time of the interviews. We are unable to draw conclusions about differences in food preferences by demographics such as race/ethnicity. Lastly, due to stigma, we may have underestimated the risk of FI in participants when asking if they could afford the type of food they enjoyed, as they were asked verbally with other community members in proximity. Furthermore, we did not screen for FI using a validated instrument.

## Conclusion

To more effectively address the unmet food needs of families at risk of FI who utilize community-based hunger relief programs, a greater understanding is needed regarding dietary and food preparation preferences. We found that families prefer similar meals, but may prepare them differently based on cultural practices among groups of people who speak English versus Spanish. Furthermore, there was little interest in learning new ways to prepare food. Taking into account that diverse, low-income communities often have limited resources, educational and food delivery interventions should be tailored to most effectively meet the needs of families.
